# Clinical and Molecular Characterization of Ataxia with Oculomotor Apraxia Patients In Saudi Arabia

**DOI:** 10.1186/1471-2350-12-27

**Published:** 2011-02-16

**Authors:** Saeed A Bohlega, Jameela M Shinwari, Latifa J Al Sharif, Dania S Khalil, Thamer S Alkhairallah, Nada A Al Tassan

**Affiliations:** 1 Department of Neurosciences, King Faisal Specialist Hospital and Research Center. P.O box 3354 Riaydh 11211 Saudi Arabia; 2 Department of Genetics, King Faisal Specialist Hospital and Research Center. P.O box 3354 Riyadh 11211 Saudi Arabia

## Abstract

**Background:**

Autosomal recessive ataxias represent a group of clinically overlapping disorders. These include ataxia with oculomotor apraxia type1 (AOA1), ataxia with oculomotor apraxia type 2 (AOA2) and ataxia-telangiectasia-like disease (ATLD). Patients are mainly characterized by cerebellar ataxia and oculomotor apraxia. Although these forms are not quite distinctive phenotypically, different genes have been linked to these disorders. Mutations in the *APTX *gene were reported in AOA1 patients, mutations in *SETX *gene were reported in patients with AOA2 and mutations in *MRE11 *were identified in ATLD patients. In the present study we describe in detail the clinical features and results of genetic analysis of 9 patients from 4 Saudi families with ataxia and oculomotor apraxia.

**Methods:**

This study was conducted in the period between 2005-2010 to clinically and molecularly characterize patients with AOA phenotype. Comprehensive sequencing of all coding exons of previously reported genes related to this disorder (*APTX*, *SETX *and *MRE11*).

**Results:**

A novel nonsense truncating mutation c.6859 C > T, R2287X in *SETX *gene was identified in patients from one family with AOA2. The previously reported missense mutation W210C in *MRE11 *gene was identified in two families with autosomal recessive ataxia and oculomotor apraxia.

**Conclusion:**

Mutations in *APTX *, *SETX *and *MRE11 *are common in patients with autosomal recessive ataxia and oculomotor apraxia. The results of the comprehensive screening of these genes in 4 Saudi families identified mutations in *SETX *and *MRE11 *genes but failed to identify mutations in *APTX *gene.

## Background

Ataxia with Oculomotor Apraxia (AOA) is an autosomal recessive cerebellar ataxia (ARCA) mainly characterized by ataxia, oculomotor apraxia and choeroathetosis [1]. Two clinically overlapping forms were characterized; AOA1 (MIM# 208920) and AOA2 (MIM# 606002). Patients with AOA1 present with cerebellar ataxia and oculomotor apraxia between ages 2 and 18 years old [2] accompanied later in life by limb dysmetria and sensory-motor neuropathy which may be associated with dystonia or mental retardation, hypoalbuminemia, hypercholesterolemia and normal immunoglobulins and alpha-fetoprotein levels. There is no evidence of chromosomal instability and no reported tumor predisposition in these patients [3]. Patients with AOA2 present with gait ataxia, cerebellar atrophy, sensory-motor neuropathy, ocular-motor apraxia and elevated immunoglobulins and alpha-fetoprotein levels with an age of onset (10-22years)[4]. In addition patients with Ataxia-Telangiectasia-Like Disorder (ATLD also known as *MRE11 *ataxia MIM# 604391) present with early onset ataxia and oculomotor apraxia [5]

Although these forms may not be quite distinctive phenotypically, they are genetically heterogeneous. Mutations in the *APTX *gene located on chromosome 9p13.3 were identified in patients with AOA1. These include; missense, nonsense, splice mutations, single base insertions and deletions [2,6-9]. A deletion of the whole *APTX *ORF was reported in one family with AOA1 phenotype [6]. Most mutations were clustered between codons 198-280. Moreover; recurrent mutations were reported in some populations; (689insT, P206L) in Japanese patients and (W279X) in Portuguese families [6]. The *APTX *gene encodes a histidine-triad (HIT) protein known as aprataxin [8].

Aprataxin is a nuclear protein of three domains; a forkhead-associated (FHA) domain that mediates protein-protein interaction with molecules that respond to DNA damage such as binding to DNA single strand break repair scaffold protein (XRCC1) and binding to DNA double strand break repair scaffold protein (XRCC4). Aprataxin also contains a histidine triad (HIT) domain and a COOH terminal zinc finger domain [7,8,10,11], the HIT domain is similar to Hint, a universal conserved AMP-lysine hydrolase, studies showed that Aprataxin has an active site dependent AMP lysine and GMP lysine hydrolase activity that also depends on the zinc finger for protein stability and on the FHA domain for enzyme activity [11].

A number of missense, nonsense and frameshift mutations in the *SETX *gene located in 9q34 were identified in patients with AOA2 and a duplication covering exons 7-10 was also identified in a patient with AOA2 [12,13]. *SETX *gene is also mutated in the autosomal dominant form of juvenile amyotrophic lateral sclerosis (ALS4) and tremor/ataxia syndrome [14,15]. Most reported mutations in AOA2 patients were clustered in exons 6 and 8 and a common hot spot mutation at codon 1363 resulting in a change from amino acid arginine to a truncating codon was recurrent in families from Portugal, Cabe Verde and Spain [12]. Senataxin, the product of *SETX *is a 2677 amino acid protein which harbors a C-terminus 7-motif domain (DNA/RNA helicase domain) found in helicases superfamily1, suggesting that senataxin my play a role in DNA repair [12,16]. Mutations in one of the MRN complex genes; *MRE11 *located in 11q21 were reported in patients with ATLD [5,17,18]. In the current study we describe the clinical phenotypes and molecular characterization of 9 patients with autosomal recessive ataxia and oculomotor apraxia from 4 unrelated consanguineous families. We identified a novel truncating mutation R2287X in *SETX *in one family in addition to the previously reported mutation W210C in *MRE11 *gene in two families. These findings emphasize on the role of these genes in such disorders.

## Methods

### Families and samples

**Figure 1:**
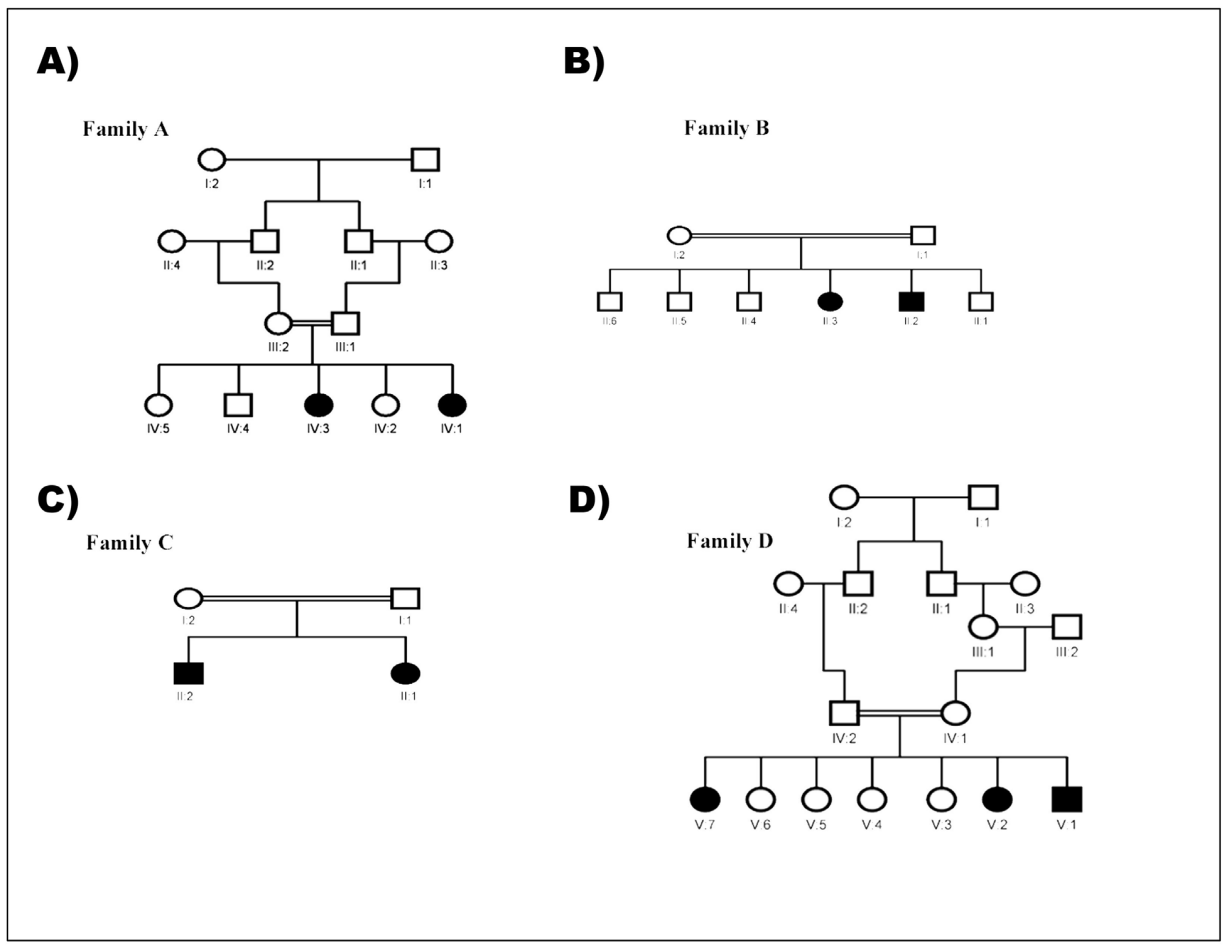
**Pedigrees of AOA1 and AOA2 families**. Each family has at least two affected individuals and autosmal recessive mode of inheritance.

This study was ethically approved (RAC#2050036) and conducted in the period between 2005-2010 at King Faisal Special hospital and Research Center (KFSHRC) which is a major tertiary care hospital in Saudi Arabia. The medical records databases were requested to provide us with all patients diagnosed with ataxia and/or oculomotor apraxia. We were able to identify and enroll four families (9 affected individuals) from the central and western regions of Saudi Arabia presented with clinical features that falls within Ataxia Oculomotor Apraxia spectrum. Consanguinity is a major feature in all these families. Clinical details of patients are summarized in Table 1 and pedigrees are presented in Figure 1. Further details on these families are described below:

**Table 1:** Clinical and biochemical characteristics of AOA patients participated in the study.

	Family A		Family B		Family C		Family D		
**Patient**	IV.1	IV3	II.2	II.3	II.1	II.2	V.1	V.2	V.7

**Sex**	F	F	M	F	F	M	M	F	F

**Age**	27	23	33	31	25	23	30	27	16

**Age of onset**	14	20	15	17	3	2	2	3	2

**Ataxia**	+++	++	++	+++	++	+	++	+++	+++

**Ocular Apraxia**	++	NP	+	+	++	+++	++	+	+

**Dysarthria**	+	NP	+	+	++	+	+	+	NP

**Mental subnormality**	NP	NP	++	++	+	+	NP	NP	NP

**Cerebellar atrophy**	++	++	NP	NP	++	++	++	++	++

**Tendon reflexes**	Absent	Absent	Normal	Normal	Absent	Absent	Absent	Absent	Absent

**Nerve conduction study**	Axonal poly.	Axonal poly.	Normal	Normal	Axonal poly.	Axonal poly.	Normal	Normal	Normal

**AFP (n 0-15)**	24.4	51.0	2.0	1.8	3.5	2.5	4.3	4.2	4.1

**Gene/Mutation**	*SETX*/R2287X		*MRE11*/W210C		No mutations in *APTX, SETX, MRE11*		*MRE11*/W210C		

M male, F female, NP Not present, + mild, ++ moderate, +++ severe

Family A: two sisters aged 27 (IV:1) and 23 (IV:3) were examined, age of onset 14 and 20 years respectively; parents were related, the other three siblings were normal. Only the elder sister had clear oculomotor apraxia, very slow horizontal saccadic ocular movement, vertical saccads were minimally affected, smooth pursuit ocular movement were poor in all directions. Frequent head thrusts were noted, tendon reflexes were absent and sensor nerve action potentials were reduced while alphaphetoprotein was elevated.

Family B: Parents were first cousins. One male aged 33 (II:2)and one female aged 31 (II:3)were examined the remaining four siblings were normal. Progressive ataxia started at ages15 and 17 years and patients were still ambulatory with difficulty at the stated age. Vertical more than horizontal oculomotor apraxia was noted with absent voluntary vertical eye movement and intact oculocephalic reflex. In addition both patients had cognitive and behavioral problem with forgetfulness, lack of attention, word finding difficulty, emotional liability, impulsivity and disinhibition with fragmented and reversed sleep patterns. Tendon reflexes and normal nerve conduction studies were normal. EEG was diffusely dysarythmic. Brain MRI showed no cerebellar atrophy.

Family C: Parents were second-degree cousins, 25 years old sister (II:1) and 23 years old brother (II:2) were examined, and one niece was similarly affected (not examined). Gait and limb ataxia were noted around 2-3 years of age, ocular apraxia started around age of 10 years, smooth pursuit ocular movement was absent in all directions and saccadic eye movement was also defective. Small saccads were substituting for the large one and required head thrusts. Brain MRI showed severe cerebellar atrophy and Brain FDG-PET showed cerebellar hypometabolism. Decreased vibration sense was noted distally, and axonal mainly sensory polyneuropathy was observed with nerve conduction study.

Family D: Parents were first cousins. Three affected individuals, 30 (V:1), 27 (V:2) and 16 (V:7) years old were examined. Five other nephews and nieces were historically affected but not studied. Ataxia was noted at age two years and oculomotor apraxia was variable among the affected individuals with very slow saccadic eye movement and impaired horizontal more than vertical pursuit eye movement with head thrusts. Vibration senses were decreased distally and tendon reflexes were absent. There was no cognitive impairment. Dystonic and choreatic movement were frequently noted. The disease was relentlessly progressive and patients became wheelchair-bound around age of 18 years. Intrafamilial variability was noticeable with the middle patient more severely affected.

### Samples

Blood samples were obtained with informed consent from family members in adherence with institutional and international guidelines (RAC#2050036). DNA was extracted from 3 ml of whole blood using Gnetra systems according to manufacturer conditions. DNA concentrations were determined.

### Amplification and sequence of *APTX*, *SETX *and *MRE11 *genes

The coding exons and flanking intronic sequences of *APTX*, *SETX *and *MRE11 *genes were amplified (primers are available upon request). PCRs were performed in a 25 μl reaction volume containing 2.5 μl of 10× reaction buffer (15 mM MgCl_2_,), 0.2 mM dNTPs, 10 μM of ARMS primers, 5 μM of IC primers, 1 U of HotStarTaq polymerase (QIAgen), and 10 ng genomic DNA. Cycling parameters were 94°C for 10 min, 30-35 cycles of 94°C for 45 sec, 52-59°C for 45 sec and 72°C for 45 sec followed by a final elongation step at 72°C for 10 min. Products were visualized on ethidium bromide stained agarose gels.

PCR products were sequenced using ABI Prism Big Dye™Terminator ready reaction cycle sequencing kit (Applied Biosystems). Products were electrophoresed using automated ABI DNA sequencer. Sequencing results were exported in one of several formats for visualization and analysis of sequence using Lasergene 6 software package.

### Microsatellite Analysis

Microsatellite markers flanking *APTX *(D9S83, D9S1788, D9253) and *SETX *(D9S1861, D9S179, D9S1847, D9S2127) were used for further genotype analysis. PCR was performed using 0.24 uM primers (forward primer FAM labeled), 2.5 mM dNTP, 10× buffer and 1 Unit of Taq polymerase and 10 ng of DNA in a 10 ul reaction. Products were run on a MegaBace 1000 capillary sequencer. Results were analyzed using MegaBase Genetic profiler V.2 (Amersham Biosciences).

### Linkage analysis

10K SNP genotyping was performed as detailed by Affymetrix on the GeneChip^® ^Human Mapping 10K Array Xba 142 2.0. In summary, 50ng of genomic DNA was digested with 10 units of *Xba*I (New England Biolabs, MA) for 2 hours at 37°C. T4 DNA Ligase was used to ligate the Xba Adaptor (Affymetrix, CA) to the digested ends. The ligated samples were diluted with water and were used as PCR templates for primers specific to the adaptor sequence with the following cycling parameters: 95°C for 3 minutes initial denaturation, 95°C 20 seconds, 62.5°C 15 seconds, 72°C 15 seconds for a total of 35 cycles, followed by 72°C for 7 minutes final extension. PCR products were then purified with salt precipitation and fragmented at 37°C for 45 minutes. The fragmented DNA was then end-labeled with biotin using 1.5 units of terminal deoxynucleotide transferase (TD) the reaction was incubated at 37°C for 2 hours. Labeled DNA was then hybridized onto the 10K Mapping Array DNA Chip at 48°C. The hybridized array was washed, stained, and scanned according to the manufacturer's instructions.

SNP genotypes were generated using affymetrix GCOS 1.4 software. Multipoint LOD score was performed with easy linkage software package.

## Results

### Mutations in *SETX *gene

**Figure 2:**
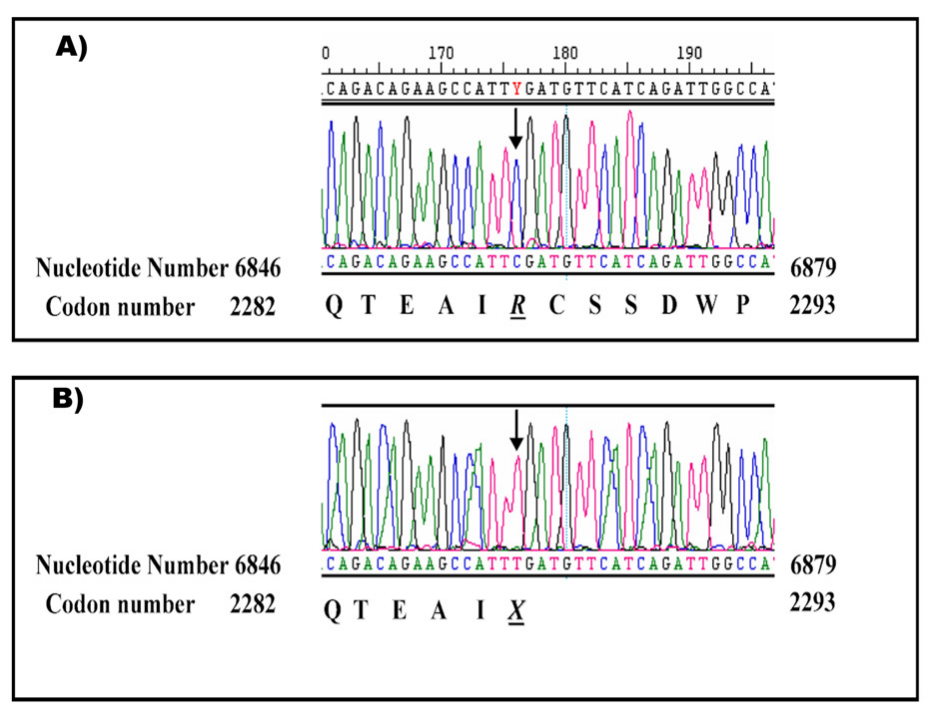
**Ssequence chromatogram showing the region of exon 20 of *SETX *gene where the R2287X (c.6859 C > T) is located**. (A) A normal control sequence trace homozygous for the normal allele (harbouring a "C" at position 6859). (B) The patient is homozygous for the mutant allele (harbouring a "T" at position 6859). Arrow points to the base substitution.

A novel truncating mutation (c.6859 C > T, R2287X) in exon 20 of the *SETX *gene was identified as the disease causing mutation in family A. All affected individuals were homozygous for this mutation while other family members were heterozygous carrying both wild-type and mutant allele (Figure 2).

### Variants Identified in *SETX *and *APTX *genes

A total of 11 missense, silent and intronic variants were identified in both genes. These include *APTX *c836+91t > c. *SETX *c846+20a > g, 846+30t > c, T1855A, D1937, T2587V and the previously reported variants in *SETX *gene Y359, H1049, D1192E, G1252R and I1385V[12]. None of these variants segregated with the disease in families B and C.

### Haplotype analysis in Family D

Haplotype results using both microsatellite and intragenic markers excluded both *APTX *and *SETX *genes as the underlying genetic cause in this family.

### Linkage analysis

linkage analysis on family D revealed a disease locus in 4 Mb regions on the long arm of chromosome 11 with a maximum multipoint logarithm of odds (LOD) score of 3.2 harboring *MRE11 *gene.

### Mutations in *MRE11 *gene

**Figure 3:**
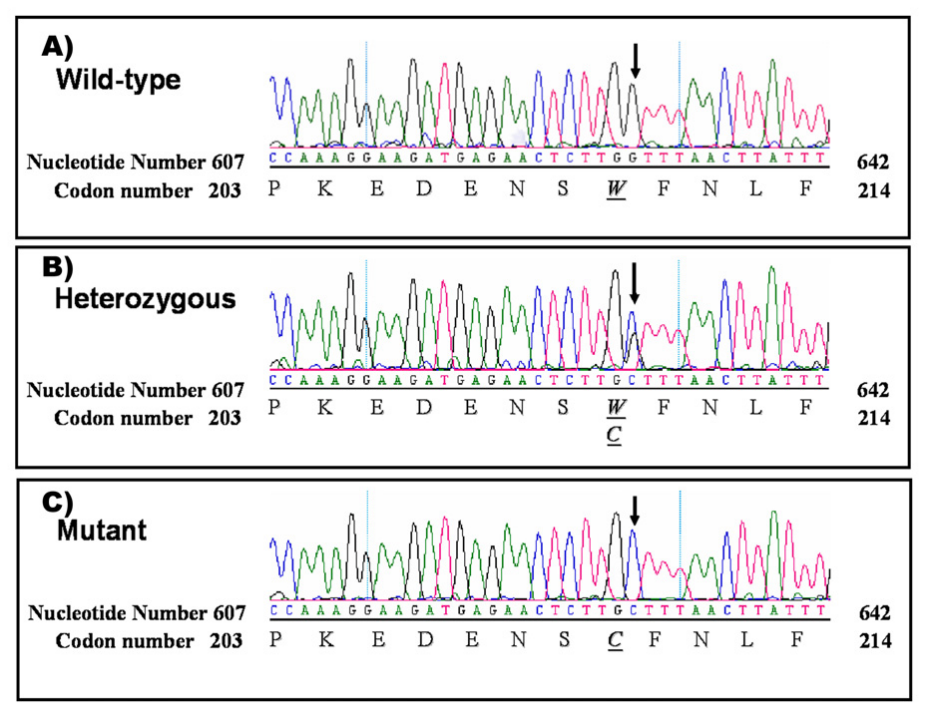
**Exon 7 of *MRE11 *gene sequence showing the location of W210C (c.630 G > C) mutation**. (A) Wild type sequence. (B) Heterozygous (C) Homozygous for the mutant allele. Arrow points to the base substitution.

Sequencing analysis identified a previously reported missense mutation in exon 7 of *MRE11 *gene (c.630 G > C, W210C) in family D. This mutation was also identified in affected individuals from family B (Figure 3).

## Discussion

Inherited diseases of the nervous system are medically and genetically heterogeneous group of abnormalities. Mutations in *LMNA, GAN1, KCC3, TDP1, APTX*, *SETX *and *MRE11 *were found to be the causes of autosomal recessive axonal neuropathies [19-21]. Ataxia with Oculomotor Apraxia type1 (AOA1), Ataxia with Oculomotor Apraxia type2 (AOA2), Ataxia with Oculomotor Apraxia type3 (AOA3), Ataxia Talengestasia (AT) and Ataxia Talengestasia Like (ATLD) represent a group of overlapping recessively inherited ataxias[22]. *APTX *and *SETX *gene mutations were identified in patients with AOA1 and AOA2 from different populations, indicating that these genes represent the common underlying genetic cause for AOA in these patients. In this study, a novel homozygous truncating mutation R2287X in *SETX *gene was identified in the 3 affected individuals in Family A. The *SETX *gene codes for senataxin, a protein that contains a DNA/RNA helicase and is involved in DNA repair.

Most reported mutations in *SETX *were present in exon 8 resulting in loss of the C- terminus motifs but this mutation is localized in exon 20 of the gene resulting in a protein that retains the C terminus helicase domain, thereby indicating the importance of other domains.

Such domains could possibly be involved in the interaction with other proteins involved in transcription and RNA processing including RNA polymeraseII, suggestive of an additional role for senataxin in the coordination of different transcription events[23].

Three Saudi families from consanguineous marriages with ataxia and oculomotor apraxia were enrolled in this study (Table 1). Although AOA1 affected individuals with mutations in *APTX *have been identified world wide; 13 individuals from 3 unrelated Tunisian family [6], 2 unrelated individuals from Germany [24], 3 unrelated Italian individuals [25], 2 American children [26] and 4 Caucasians with ataxia and CoQ10 deficiency [27], yet comprehensive screening by direct sequencing of the *APTX *gene did not identify any mutations in these 3 Saudi families. Microsatellite analysis further excluded this gene as disease causing in these families. However, linkage analysis on family D with 3 affected individuals identified a locus harboring *MRE11 *gene which is mutated in ATLD where there is progressive cellular degeneration, ocular apraxia and absence of ocular telangiectasia [17,18,28]. Sequencing of *MRE11 *in family D members identified the previously reported W210C missense mutations in all affected individuals from both families B and D. W210C missense mutation was identified in 10 Saudi patients with ATLD [5]. Some studies suggested that the signaling related function of the MreII/Rad50/Nbs1 (MRN) complex is related to the ATM function in mammalian cells, as certain components of the MRN complex act substrates for ATM which is in turn important for S phase checkpoint activation and cell survival following double-strand breaks [29]. Screening all these genes in affected individuals of family C failed to detect any pathogenic segregating mutation, however, we didn't check for regulatory mutations or large deletions. Moreover, the two affected siblings in this family had ataxia and oculomotor apraxia with no cerebellar atrophy and normal tendon reflexes and they may belong to a similar overlapping form of ataxia. None of our patients has features suggestive of ataxia telangiectasia. Particularly there was no repeated infection, tumor appearance or changes in immunoglobulins in these families.

We screened nine affected individuals from 4 families with ataxia and oculomotor apraxia for mutations in the reported genes *APTX*, *SETX *and *MRE11 *and identified a novel truncating mutation in *SETX *gene in one family and a previously reported missense mutation in *MRE11 *gene in two families.

## Conclusion

Identification of a novel truncating mutation in *SETX *gene in a Saudi family with AOA2 and the common reported mutation W210C in *MRE11 *in two families with ataxia and oculomotor apraxia supports the involvement of these genes in the disease progression. Lack of mutations in *APTX*, *SETX *and *MRE11 *genes in the third family diagnosed with ataxia and oculomotor apraxia and no cerebellar atrophy suggests the involvement of another mechanism for the development of this disorder.

## Competing interests

The authors declare that they have no competing interests.

## Authors' contributions

SB & TK were responsible for patient's phenotyping and sample collection. JS, LS, DK participated in gene amplification and sequence analysis, NT and SB participated in the design of the study. NT drafted the manuscript. Both SB and JS participated in editing the manuscript and contributed equality to the manuscript. All authors read and approved the manuscript.

## Pre-publication history

The pre-publication history for this paper can be accessed here:

http://www.biomedcentral.com/1471-2350/12/27/prepub
